# Segregation analysis of the *BRCA2* c.9227G>T variant in multiple families suggests a pathogenic role in breast and ovarian cancer predisposition

**DOI:** 10.1038/s41598-020-70729-0

**Published:** 2020-08-19

**Authors:** Simona Agata, Silvia Tognazzo, Elisa Alducci, Laura Matricardi, Lidia Moserle, Daniela Barana, Marco Montagna

**Affiliations:** 1 Immunology and Molecular Oncology Unit, Veneto Institute of Oncology IOV-IRCCS, Padua, Italy; 2 Oncology Unit, Local Health and Social Care Unit ULSS8 Berica, Montecchio Maggiore, Italy

**Keywords:** Breast cancer, Medical genetics

## Abstract

Classification of variants in the *BRCA1* and *BRCA2* genes has a major impact on the clinical management of subjects at high risk for breast and ovarian cancer. The identification of a pathogenic variant allows for early detection/prevention strategies in healthy carriers as well as targeted treatments in patients affected by *BRCA*-associated tumors. The *BRCA2* c.9227G>T p.(Gly3076Val) variant recurs in families from Northeast Italy and is rarely reported in international databases. This variant substitutes the evolutionary invariant glycine 3076 with a valine in the DNA binding domain of the BRCA2 protein, thus suggesting a high probability of pathogenicity. We analysed clinical and genealogic data of carriers from 15 breast/ovarian cancer families in whom no other pathogenic variants were detected. The variant was shown to co-segregate with breast and ovarian cancer in the most informative families. Combined segregation data led to a likelihood ratio of 81,527:1 of pathogenicity vs. neutrality. We conclude that c.9227G>T is a *BRCA2* pathogenic variant that recurs in Northeast Italy. It can now be safely used for the predictive testing of healthy family members to guide preventive surgery and/or early tumor detection strategies, as well as for PARP inhibitors treatments in patients with *BRCA2*-associated tumors.

## Introduction

Next to the hurdle of the bioinformatics processing of huge amount of sequencing data, the clinical interpretation of sequence variants has become the most recent challenge of next generation sequencing (NGS) approaches. Efforts are currently underway within international consortia such as the Evidence-based Network for the Interpretation of Germline Mutant Alleles (ENIGMA) to order and standardize a variety of methods that foster variants of uncertain significance (VUS) towards a benign or pathogenic classification.

While pathogenic variants of the *BRCA1* and *BRCA2* genes account for about one fourth of all breast and ovarian cancer families^1^, VUS are the result of a smaller fraction of all tests (2–20%)^2,3^ and cannot be used for identification of predisposed family members as long as their clinical relevance is clearly defined. In particular, predictive testing within families is only recommended for variants with a probability of pathogenicity higher than 95% (i.e. class 4 and 5 according to a widely used 5-tiered classification)^4^. In the absence of a pathogenic variant, healthy subjects of high risk families need to be managed according to the specific family history of the disease.

Probabilities of pathogenicity for variants occurring in the *BRCA1* and *BRCA2* genes were previously calculated based on variant location within splicing consensus sequences^5^ or cross-species evolutionary conservation of each aminoacid positon^6^. These estimates were calibrated against large clinical data sets to generate a priori probabilities of pathogenicity (reviewed in^7^), thus providing a hint for identification of those variants that might deserve further investigation.

On the other hand, it has been suggested that additional proofs, relying on “direct evidences”^8^, are necessary to reach a final (posterior) probability that fosters the variant from class 3, including VUS^4^, to one of the extreme classes. Using the multifactorial likelihood model, several types of data sources can contribute to variant classification, including family history of cancer, co-occurrence (*in trans*) with known pathogenic variants, breast cancer histopathological features and segregation^9^. Breast cancer histopathology provides little predictive power for *BRCA2* variants, as *BRCA2*-associated and non-hereditary breast tumors display largely overlapping morphological and biochemical parameters^10^. Similarly, co-occurrence with proven pathogenic variants is strongly predictive of neutrality. Conversely, in the absence of pathogenic variants, it provides scant evidence for a classification towards pathogenicity. Therefore, at present, the analysis of segregation of the variant with disease within families remains one of the most powerful and robust method to achieve a successful classification for class 3 *BRCA2* variants^11^.

## Results and discussion

During the molecular analysis of *BRCA1*/2 genes in more than 6,000 breast and/or ovarian cancer patients, we identified 15 families (0.25%) carrying the *BRCA2* c.9227G>T variant. All families were selected according to criteria approved by the Veneto Region and largely overlapping to those currently used in European countries (see “Methods” section for details). Most of the families carrying the *BRCA2* c.9227G>T variant showed typical *BRCA2* tumor spectra with frequent bilateral breast tumors, early age at first breast cancer diagnosis, and presence of ovarian cancer in more than half of them (Table 1).

**Table 1:** Characteristics of families carrying the *BRCA2* c.9227G>T.

Family ID	Number of breast cancer cases (age)	Number of ovarian cancer cases (age)	Number of breast cancer phenocopies (age)	Other tumors (age)	BOADICEA	Number of other tested family members	LR^a^
33	6 (**50**, 44**, 32/40**^b^, 30, 52, 41)	–	–	Kidney (**65**)	92.2	1	3.44
72	4 (**42/46**^b^, 53, 59, 33)	–	1 (**60**)	–	71.2	1	1.17
139/2102	3 (**47**, **47**, **48**)	1 (66)	–	Central nervous system (53)	3.9	2	3.7
219	5 (**44**, 37, **50**^c^, **50**, **65**)	1 (**57**^c^)	1 (**55/55**^**b**^)	Kidney (**63**), lung (**66**)	52.9	3	10.77
716	2 (**59**^c^, 24)	1 (**53**^c^)	–	–	73.3	3	1.03
873/987	6 (**50**, **44**, 25, **49/58**^b^, 33, **81**)	2 (**59**, 63)	1 (**55**)^d^	–	93.2	8	40.61
1339	2 (**40, 44**)	–	–	–	3.6	1	1.87
1579	2 (**31**, **56**)	–	–	Ampulla of Vater (**60**)	10.4	4	3.16
2535	3 (**45**, 45/78^b^, 43)	–	–	–	20.0	–	–
2848	2 (**37**, 46)	1 (62)	1 (**42**)	–	31.5	1	1.13
3336	3 (**43**^c^, **60**, 46)	1 (**55**^c^)	–	Kidney (61), prostate (75)	33.0	1	1.82
3816	2 (55/70^b^, 50)	1 (**68**)	–	–	19.0	–	–
4196	1 (**43**)	1 (**66**)	–	Prostate (44), thyroid (64)	8.4	–	–
4482	2 (**35**, 40)	–	–	Pancreas (63)	7.6	–	–
5405	1 (65)	1 (**47**)	–	–	2.9	–	–

Numbers in bold refer to age at diagnosis in individuals specifically analysed for the c.9227G>T or obliged carries.

^a^*LR* likelihood ratio.

^b^Bilateral breast cancer.

^c^Subjects affected by breast and ovarian cancer.

^d^*LCIS* lobular carcinoma in situ.

Based on family histories of breast and ovarian cancer, a high probability of occurrence of a *BRCA1* or *BRCA2* pathogenic variant was obtained in most of the families (Table 1). In spite of these predictions, neither clearly pathogenic variants nor other VUS were identified in addition to the *BRCA2* c.9227G>T in any of these families. Although screening of *BRCA1* and *BRCA2* genes was performed by different technical approaches over the time, it always included the complete coding sequence as well as all exon–intron boundaries of both genes, thus minimizing the possibility that pathogenic variants in *BRCA1* or located *in cis* to the *BRCA2* c.9227G>T variant might have been missed. Since the analysis included only the *BRCA1* and *BRCA2* genes, the presence of pathogenic variants in other high/moderate predisposition genes could not be excluded.

Glycine 3076 amino acid is an invariant position across twelve species from *Pan troglodytes* to *Strongylocentrotus purpuratus* (see “Methods” section for the complete list of species). Comparison of the composition, polarity and molecular volume of glycine vs. valine, highlights a moderate physicochemical difference corresponding to a Grantham distance^12^ of 109. Using Align-GVGD^13,14^, a widely used in silico prediction tool, the combination of these features assigns this aminoacid substitution to category C65, which includes the most likely deleterious changes. Glycine 3076 is located within the oligonucleotide binding-3 motif (OB-3) of a larger domain specifically involved in ssDNA binding^15^. Altogether these data strongly favour a likely functional relevance of the p.(Gly3076Val) substitution. According to previous estimates^6^, these observations provide a 0.81 a priori probability of pathogenicity.

The DNA binding motif is the most characterized functional domain of the BRCA2 protein and has been implicated in the homologous recombination activity necessary for the repair of DNA double strand breaks. The relevance of the domain is emphasized by the high density of pathogenic missense variants mapping to this motif. Accordingly, using a homology-directed DNA break repair (HDR) functional assay, Guidugli et al.^16^ showed that 18 of 33 (54%) VUS located in this domain displayed an impaired ability to repair an I-Sce1-induced DNA double strand break. In particular, when challenged by this method, p.(Gly3076Val) showed an in vitro phenotype overlapping to those of pathogenic variants^16^. Using the classification guidelines from the American College of Medical Genetics (ACMG)^17^, data derived from well-established functional studies provide a strong evidence of pathogenicity (PS3 category). This feature, combined with the absence of the c.9227G>T variant in control populations (PM2), and with the in silico evidence of pathogenicity (PP3), would move the variant to class 4 (likely pathogenic). However, it has to be noted that different functional assays, though extremely powerful in some contexts, can lead to inconsistencies depending on the specific experimental conditions. Moreover, they often lack proper validation in terms of sensitivity and specificity. While efforts are currently in progress within the ENIGMA consortium to derive rules to include functional assays results into the multifactorial likelihood model^18^, at present further evidences are advisable to derive a final probability of pathogenicity to confidently support clinical management decisions.

We therefore evaluated segregation of c.9227G>T in a total of 25 additional family members from 10 of the 15 families. Likelihood scores were calculated by means of a co-segregation algorithm specifically designed for the evaluation of *BRCA1* and *BRCA2* class 3 variants^19^. In addition to genotypes, this method makes use of age of onset (with penetrance used as a function of age) of first and second breast cancer as well as ovarian cancer. Based on the assumption of independence of all sources of evidence that are integrated into the multifactorial likelihood method, “family history” data were not used further in the analysis. Co-segregation likelihood ratios are reported in Table 1 for families with at least 2 family members genotyped. Very similar results were obtained when the most informative families were evaluated by an alternative full likelihood Bayes factor algorithm^11^ (data not shown).

**Figure 1:**
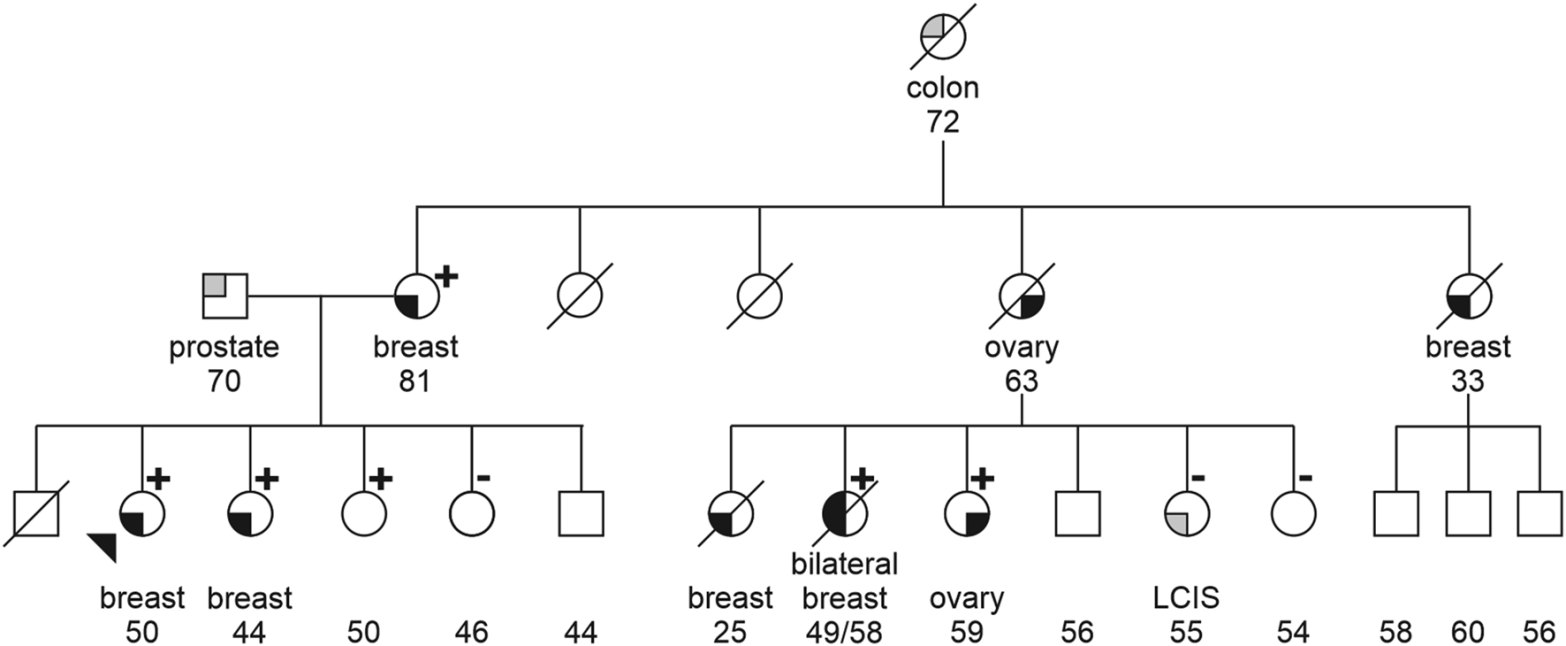
Segregation of the *BRCA2* c.9227G>T in family 873/987. Carriers and non carriers are indicated by + and − signs, respectively. Tumor type is indicated below each symbol. Numbers refer to current age and age at diagnosis for healthy and affected subjects, respectively. Proband is marked by the arrow. *LCIS* lobular carcinoma in situ.

A combined likelihood ratio of 81,527:1 was obtained from the integration of all family scores, generating a probability of pathogenicity higher than 99.9%, that definitely assigns this variant to class 5. Segregation of c.9227G>T with disease was nearly complete, with few exceptions. In family 873/987 the proband’s maternal cousin was negative for c.9227G>T and developed a lobular carcinoma in situ (LCIS) at age 55 (Fig. 1). LCIS, however, has gradually moved from a rare form of breast cancer to a “marker” of increased cancer risk and it is commonly referred to as “lobular neoplasia”. As such, it is not usually taken into consideration in computer modelling of mutation probability; accordingly, it was excluded from the calculation of the segregation likelihood ratio (i.e. this subject was treated as a healthy one). In contrast, three phenocopies in family 72, 219 and 2848 were included in score calculations. These three patients were third degree relatives of the closest carrier, with 1–2 healthy subjects interposed, and at least two of them had a positive family history in the alternative parental branch. Because of the genealogic distance from the proband, likelihood ratios were only marginally lowered by these data and remained in favour of pathogenicity in each of these families.

Considering all carrier family members, mean age at first breast and ovarian cancer were 48 and 60 years, respectively, consistent with those reported for *BRCA2* pathogenic variants^20^. Similarly, the ratio of breast to ovarian cancer was in line with what expected for a pathogenic variant falling outside of the breast cancer cluster region (BCCR) and the ovarian cancer cluster region (OCCR)^21^.

Among the other tumors anecdotally reported as part of the *BRCA2* spectrum, an ampulla of Vater carcinoma occurred in a carrier from family 1579. Increased risk of gallbladder and bile duct tumors were initially observed among *BRCA2* carriers^22^. Interestingly, recent data apparently reinforce the association of the *BRCA2* gene to this specific tumor type^23,24^. Considering the rarity of the tumor it might represent a good predictor of pathogenicity for *BRCA2* variants, especially when associated with a family history of breast and/or ovarian and/or pancreatic cancer. Renal cell cancers were observed in three subjects from independent families, two of whom were obliged carriers of the *BRCA2* variant. Though a role for *BRCA2* has been suggested in the kidney embryonic development of the zeppelin zebrafish mutant^25,26^, renal cancer is only sporadically reported among *BRCA2* carriers. Therefore, unless of a variant-specific effect, the cases reported here remain a descriptive observation.

With four entries in the ClinVar database^27^ during the last 16 years (record VCV000126203.3, accessed on July 10, 2020), the c.9227G>T variant has never been reported in international population databases such as “The Genome Aggregation Database” (https://gnomad.broadinstitute.org/), thus suggesting a geographically limited distribution with a higher prevalence in the Veneto Region of Italy. This observation has important implications for sequence variants classification, as the power of segregation analysis increases with the number of families studied. While benign classification of commonly identified variants is more easily achieved by laboratories with a high throughput (the large amount of tests increases the probability of identification of a co-occurrence with pathogenic variants), local laboratories might retain a higher classification power for specific variants with a peculiar geographical distribution.

Of note, the proband of family 1579 developed an invasive ductal breast cancer at age 31 that progressed to a metastatic disease four years later. She was therefore proposed a *BRCA* test in the context of a large clinical trial to apply for a PARP-inhibitor treatment. The *BRCA* test was centralized outside our Institute; she turned out to be a carrier of the c.9227G>T “variant of uncertain significance” and she was therefore excluded from the trial. This emphasizes the different consequences related to the inability to classify a VUS. Indeed, while in a family context this failure implies that all at risk subjects need to be “included” into tailored surveillance strategies, based on their family history, this inability can represent an “exclusion” criterion from specific treatments for the patient. Importantly, the list of BRCA-associated tumors that can benefit from PARP inhibitors treatment is rapidly growing and it now includes: metastatic Her2-negative breast cancer, metastatic pancreatic cancer and metastatic castration-resistant prostate cancer, in addition to high grade ovarian cancers^28^.

In conclusion, our data demonstrate that the *BRCA2* c.9227G>T variant co-segregates with disease in multiple families and shows a phenotypic expression falling within the classical *BRCA2*-associated spectrum. These findings, combined with in silico predictions as well as functional impairment of the DNA double strand break repair, provide definitive evidence for pathogenicity, thus reliably moving the variant to class 5 (definitely pathogenic). The *BRCA2* c.9227G>T variant can therefore be safely used in families to identify predisposed family members and to guide risk-reducing surgery as well as strict surveillance strategies. Concurrently, patients carrying the *BRCA2* c.9227G>T variant can benefit from targeted treatments of PARP-inhibitors sensitive tumors.

## Methods

Sequence variants are described according to HGVS nomenclature guidelines (https://varnomen.hgvs.org/) and the *BRCA2* Refseq NM_000059.3.

Families were identified during the molecular analysis of *BRCA1* and *BRCA2* genes offered to patients with personal and/or family history of breast and/or ovarian cancer according to selection criteria approved from the Veneto Region. Briefly, referral criteria included (a) a personal history of either of the following: breast cancer before age 36, bilateral breast cancer before age 50, male breast cancer, breast and ovarian cancer in the same patient, triple negative breast cancer (i.e. negative for estrogen receptor, progesterone receptor, and HER2) before age 60, high grade ovarian cancer; or (b) a family history including: (i) two first degree relatives with bilateral breast cancer and/or breast cancer before age 50 or (ii) three first degree relatives affected by breast and/or ovarian and/or pancreatic cancer.

The search for pathogenic variants was carried out on DNA extracted from peripheral blood. Direct sequencing, either Sanger sequencing or NGS (Illumina MiSeq platform), was used for the vast majority of the probands. Major genomic rearrangements were analysed by multiplex ligation-dependent probe amplification (MLPA) or NGS-based approaches (Sophia DDM, Sophia Genetics). Only the specific variant under study was tested in the other family members.

In silico predictions were performed by means of the Align-GVGD program^13,14^, freely available at https://agvgd.hci.utah.edu/. Calculations were made using the largest number of alignments including the following species: *Homo sapiens, Pan troglodytes, Macaca mulatta, Rattus norvegicus, Canis familiaris, Bos taurus, Monodelphis domestica, Gallus gallus, Xenopus laevis, Tetraodon nigroviridis, Fugu rubripes, *and* Strongylocentrotus purpuratus*.

All procedures were in accordance with the ethical standards of the 1964 Helsinki declaration and its later amendments. Probands and family members who were tested for the *BRCA2* c.9227G>T explicitly agreed to participate to the research project and signed an informed consent. All experimental protocols were approved by the Ethics Committee of the Veneto Institute of Oncology IOV.

Probabilities to identify a pathogenic variant were computed using the breast and ovarian analysis of disease incidence and carrier estimation algorithm (BOADICEA)^29^.

Current age, gender, age of onset of the first and second breast cancer, age of onset of ovarian cancer and genotype of members of families carrying the *BRCA2* c.9227G>T variant were used to calculate likelihood ratios of the variant to be pathogenic vs. neutral using an approach previously described for *BRCA1* and *BRCA2* variant co-segregation analysis^19^. Families with the highest pathogenicity likelihood were double-checked using an alternative full likelihood Bayes factor approach available at https://analyze.myvariant.org/cosegregation-analysis^11^.

The overall likelihood was derived by the product of the likelihood ratios over the independent families.
